# Effective Removal of Staphylococcal Biofilms by the Endolysin LysH5

**DOI:** 10.1371/journal.pone.0107307

**Published:** 2014-09-09

**Authors:** Diana Gutiérrez, Patricia Ruas-Madiedo, Beatriz Martínez, Ana Rodríguez, Pilar García

**Affiliations:** Instituto de Productos Lácteos de Asturias (IPLA-CSIC), Villaviciosa, Asturias, Spain; Universitätsklinikum Hamburg-Eppendorf, Germany

## Abstract

Staphylococcal biofilms are a major concern in both clinical and food settings because they are an important source of contamination. The efficacy of established cleaning procedures is often hindered due to the ability of some antimicrobial compounds to induce biofilm formation, and to the presence of persister cells, a small bacterial subpopulation that exhibits multidrug tolerance. Phage lytic enzymes have demonstrated antimicrobial activity against planktonic and sessile bacteria. However, their ability to lyse and/or select persister cells remains largely unexplored so far. In this work, the lytic activity of the endolysin LysH5 against *Staphylococcus aureus* and *Staphylococcus epidermidis* biofilms was confirmed. LysH5 reduced staphylococcal sessile cell counts by 1–3 log units, compared with the untreated control, and sub-inhibitory concentrations of this protein did not induce biofilm formation. LysH5-surviving cells were not resistant to the lytic activity of this protein, suggesting that no persister cells were selected. Moreover, to prove the lytic ability of LysH5 against this subpopulation, both *S. aureus* exponential cultures and persister cells obtained after treatment with rifampicin and ciprofloxacin were subsequently treated with LysH5. The results demonstrated that besides the notable activity of endolysin LysH5 against staphylococcal biofilms, persister cells were also inhibited, which raises new opportunities as an adjuvant for some antibiotics.

## Introduction

Two staphylococcal species, *Staphylococcus aureus* and *Staphylococcus epidermidis*, are the main cause of hospital-associated infections [1], [2]. *S. epidermidis* is a common etiological agent of nosocomial infections, mostly occurring in immune compromised patients with implanted medical devices [3]. Methicillin-resistant *S. aureus* (MRSA) strains are also responsible for both health care and community-associated infections, mostly involving skin and wound infections, pneumonia, severe sepsis and endocarditis [4]. In addition, *S. aureus* is one of the major bacterial agents causing foodborne diseases in humans due to the production of enterotoxins [5].

Pathogenicity of both species is clearly associated with their ability to form biofilms on biotic and abiotic surfaces, providing high resistance to host defenses and antibiotics, and also to cleaning and disinfection processes [2]. The ability to form biofilms allows *S. aureus* to survive in hostile environments, such as food industry surfaces [6], and this enhances the recurrence of food contamination. The ability to form biofilms in both species is due to the production of an extracellular material that contributes to intercellular aggregation and attachment to surfaces. In most cases, this matrix is composed of exopolysaccharides such as poly-N-acetyl-β-(1,6)-glucosamine (PIA/PNAG), teichoic acids, DNA and specific proteins [2]. *S. aureus* strains that are not reliant on polysaccharides for biofilm formation have also been identified. Specific proteins such as Bap [7], Spa [8], FnBPA, FnBPB [9], and SasG [10], are surface adhesins involved in biofilm development. In *S. epidermidis* an extracellular matrix-binding protein (Embp) is necessary and sufficient to promote protein-dependent biofilm formation [11]. In addition, the accumulation-associated protein (Aap) also mediates intercellular and surface adhesion and is considered the most important factor contributing to protein-dependent biofilm formation in *S. epidermidis* [12].

Bacteria embedded in biofilms are considerably less susceptible to antibiotics than their planktonic counterparts, mainly due to the limited access which the antibiotic has into the biofilm [13]. Moreover, several studies have shown that *S. aureus* biofilm formation can be stimulated by sub-inhibitory concentrations (sub-MICs) of some antibiotics [14]. Some bacteria within the biofilm also show a reduced susceptibility to antibiotics due to their dormant phenotype. These bacteria, named persister cells, are genetically identical to susceptible bacteria and give rise to a new sensitive population after the removal of antibiotic pressure [15]. Persistance is a main cause of concern as it has been observed in most bacterial species, in relation to different classes of antibiotics, with persister cells being involved in recurrent infections [16]. In this regard, several strategies for the clearance of staphylococcal biofilms, other than disinfectants and antibiotics, have been assayed. These include PIA-degrading enzymes like dispersin B [17], the peptidoglycan-degrading enzyme lysostaphin [18], bacteriocins [19] and bacteriophages [20]–[23].

Recently, phage lytic proteins (endolysins and virion-associated peptidoglycan hydrolases) have shown a potent antimicrobial activity against planktonic bacteria [24], [25]. They have been investigated as therapeutic agents and biopreservatives against a range of pathogens, such as *Bacillus anthracis*, *Streptococcus pneumoniae*, *S. aureus*, and *Streptococcus suis*. Moreover, *S. aureus*, *S*. *suis*, *S. pneumonia* and *Streptococcus pyogenes* biofilms have been successfully removed by endolysins [26]–[29]. We have previously identified and characterized the endolysin LysH5 encoded by the *S. aureus* phage vB_SauS-phiIPLA88, which showed a high degree of similarity (97%) with the endolysin from phage phi11, for which anti-biofilm activity has previously been reported [30]. LysH5 has two catalytic domains (CHAP domain and amidase-2 domain) and a cell wall binding domain (SH3b). This protein is able to lyse a wide range of staphylococci, including bovine and human *S. aureus* and *S. epidermidis* [31]. A synergistic antimicrobial activity was previously observed with both the bacteriocin nisin [32] and the virion-associated peptidoglycan hydrolase HydH5 and its derivative fusion proteins [33].

The aim of this study was to determine the efficacy of the endolysin LysH5 to remove the biofilms formed by *S. aureus* and *S. epidermidis* strains. We have studied the ability of LysH5 to induce biofilms, to select persister cells, and finally, to see if LysH5 was also able to kill persister cells previously selected by antibiotics.

## Material and Methods

### Bacterial strains, growth media and proteins

Staphylococcal strains (Table 1) were routinely cultured in TSB broth (Tryptic Soy Broth, Scharlau, Barcelona, Spain) at 37°C with shaking, or in TSB plates containing 2% (w/v) bacteriological agar (TSA).

**Table 1:** Strains used in this work, origin and relevant properties.

	Strain	Origin	Relevantproperties	Reference
*S. aureus*	15981	Clinical isolate	Produces PNAG	[42]
	ISP479r	Clinical isolate		
	V329	Bovine subclinical mastitis	Expresses*bap*protein	[7]
	132	Clinical isolate	Produces PNAG	[43]
	IPLA1	Dairy industry surface	Genes *icaA* and *icaD*	[6]
	IPLA16	Meat industry surface		
	SA113	-	NCTC8325 derivative, agr–, 11-bp deletion in *rsbU*	[44]
	Newman	-	Sub-inhibitory concentrations of meticillin induces biofilm formation.	[45]
*S. epidermidis*	B	Breast milk of women suffering infectious mastitis	Genes *icaA* and *icaD*	[46]
	YLIC17			
	DG2n			

LysH5 purification was performed as previously described [32]. The LysH5 specific activity against the different strains was calculated as the ΔOD per mg of protein per min [31]. Lysostaphin was obtained from Sigma (Sigma, Missouri, USA).

### Biofilm assays

For biofilm formation the protocol of Herrera et al., (2007), was used with some modifications [34]. Briefly, overnight (o/n) staphylococcal cultures were diluted in fresh TSBg (TSB supplemented with 0.25% w/v D-(+)-glucose) up to 10^6^CFU/ml, and 200 µl were poured into TC Microwell 96U w/lid nunclon D SI plates (Thermo Scientific, NUNC, Madrid, Spain), and incubated at 37°C for 24 h. Wells were washed twice with sterile phosphate-buffered saline (PBS buffer) (137 mM NaCl, 2.7 mM KCl, 10 mM Na_2_HPO_4_ and 2 mM KH_2_PO_4_; pH 7.4).

To determine the counts of bacteria attached to each well once the biofilm was developed, the well was scratched twice with a sterile swab and then immersed into 9 ml of PBS buffer. A vigorous shaking for 1 min allowed the disaggregation of the biofilm [34]. Finally, several decimal dilutions were plated onto TSA and incubated at 37°C.

Likewise, the biofilm that had adhered to the surfaces of the wells was observed by staining with crystal violet (0.1% w/v) for 15 min, followed by a gentle wash with water and de-staining in acetic acid (33% v/v). Absorbance was measured at a wavelength of 595 nm [9]. All the assays were performed using two biological replicates.

### Treatment of staphylococcal biofilms with LysH5

24 h-old biofilms were developed as described previously. After the washing step with PBS buffer, 0.15 µM of LysH5, 0.2 µM of lysostaphin, or 200 µl of sodium phosphate buffer (50 mM pH 7) for control purposes, were added to each well. Plates were incubated for 6 h at 37°C and cell counts were measured as described above. Surviving cells were recovered after treatment by scratching the well with a sterile swab and transferred to 2 ml of TSB medium. For two-step treatment assay, 24 h-old biofilms were first treated with LysH5 (0.15 µM) for 6 h, then washed and treated again with LysH5 (0.15 µM) for 12 h. Cell counts were measured as described above.

### Determination of minimal inhibitory concentration (MIC) of LysH5 and biofilm induction

The MIC of LysH5 was determined in duplicate by a conventional broth microdilution technique in TSB. Two-fold dilution of LysH5 (0.4 µM) was made in microtiter plates and each well was inoculated with 10^6^ CFU. The MIC was defined as the lowest protein concentration that inhibited visible bacterial growth after 24 h of incubation at 37°C.

The ability of LysH5 to induce the formation of biofilms was tested using sub-inhibitory concentrations of the protein, ranging from 0 to 6 µg/ml (0–0.1 µM) for *S. aureus* 15981, *S. aureus* 132, *S. aureus* IPLA1, *S. aureus* IPLA6 and *S. epidermidis* YLIC17; and from 0 to 3 µg/ml (0–0.05 µM) for *S. aureus* ISP479r, *S. aureus* V329, *S. epidermidis* B and *S. epidermidis* DG2n. *S. aureus* Newman treated with 0–10 µg/ml of methicillin was used as a positive control of biofilm formation under sub-inhibitory concentrations of this antibiotic [14]. The biofilm induction value for each strain (expressed as relative absorbance) was defined as the absorbance value measured after crystal violet staining at each concentration of LysH5, or methicillin, divided by the absorbance value in the absence of the antimicrobials. For CFU determination of non-adhered cells, planktonic cells of each well were diluted and plated onto TSA plates. All experiments were conducted using two biological replicates.

### Biochemical characterization of the biofilm matrix

24 h-old staphylococcal biofilms were washed with PBS and then treated for 1 h 37°C either with a solution of 10 mM sodium metaperiodate in 50 mM sodium acetate buffer (pH 4.5) (sodium metaperiodate treatment is used to disrupt the extracellular polysaccharides), with 100 µg/ml of proteinase K (Sigma, Madrid, Spain) in 20 mM Tris (pH 7.5) and 100 mM NaCl, or with 100 µg/ml of DNAseI (Sigma, Madrid, Spain) in 150 mM of NaCl and 1 mM CaCl_2_. After treatments, the biofilms were washed with water, stained with crystal violet, and the absorbance measured as described above.

### LysH5 activity against persister cells

To determine if treatment of planktonic cells with LysH5 yielded a subpopulation of persister cells, overnight cultures of *S. aureus* 15981 were used to inoculate TSB fresh medium and grown at 37°C up to OD_600_ = 0.5. Then, LysH5 was added at 1 to 5-fold MIC (0.1–0.5 µM, respectively) and cultures were incubated at 37°C for 3 h.

Lytic activity of LysH5 against previously selected persister cells was also tested as follows. Persister cells of *S. aureus* SA113 were selected as described previously [35] after the treatment of exponential cultures (OD_600_ = 0.5) with 2 µg/ml of rifampin or 3 µg/ml of ciprofloxacin (100 and 10-fold MIC, respectively) for 4 h. LysH5 was then added at 0.5 µM and incubated at 37°C for 4 additional hours.

For CFU determinations, samples were taken before and during the antimicrobial challenge and appropriate dilutions plated onto TSA plates. All experiments were conducted using two biological replicates.

### Low-temperature scanning electron microscopy (LTSEM)

LTSEM was used to visualize the structure of the 24 h-old biofilms before and after treatment with the endolysin LysH5. The biofilms were grown on a glass cover lid and visualized at −135°C with a DSM 960 Zeiss scanning electron microscope as previously described [36].

### Statistical analysis

Statistical analysis was performed in order to establish any significant differences between the total bacteria number in the control and treated biofilms, and between the control and treated planktonic cultures. The differences were expressed as the mean ± standard error and were determined by one-way analysis of variance (ANOVA) and the LSD test was used for a comparison of means at a level of significance *P<0.05*.

## Results

### LysH5 is effective against staphylococcal biofilms

**Figure 1:**
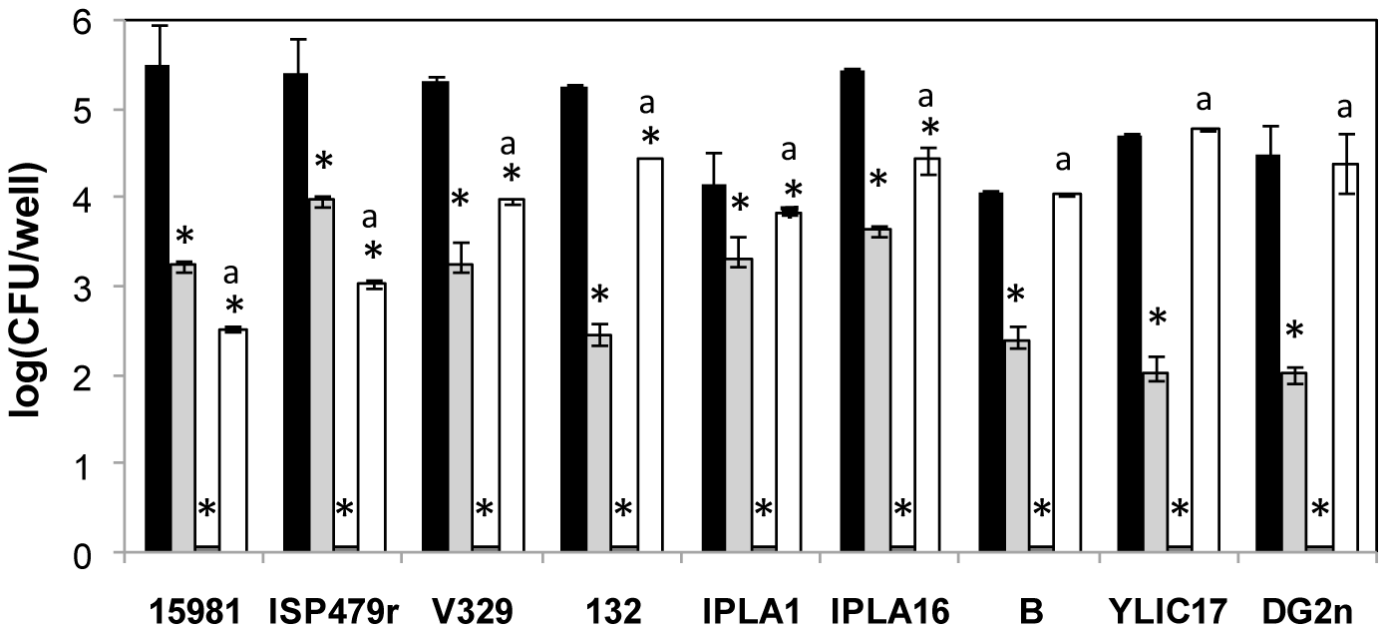
Removal of 24 h-old biofilms of *S. aureus* and *S. epidermidis*. Biofilms of *S. aureus* 15981, ISP479r, V329, 132, IPLA1 and IPLA16 and *S. epidermidis* B, YLIC17and DG2n were treated with 0.15 µM of LysH5 (light grey) and 0.2 µM of lysostaphin (white) during 6 hours. Alternatively, biofilms were treated with 0.15 µM of LysH5 during 6 hours, followed by another treatment with the endolysin for 12 hours (gross line in X axis). Control biofilms are represented in black. Adhered cell counts were expressed as log CFU/well. Bacteria detection threshold (<10 CFU/ml). Means and standard deviations were calculated from two biological replicates. Bars having an asterisk are significantly different from the control (ANOVA; *P*<0.05) and bars with a lower case ‘a’ indicates a significantly difference between the lysostaphin treatment and the treatment with LysH5 (ANOVA; *P*<0.05).

The ability of LysH5 to remove staphylococcal biofilms and kill sessile cells was determined on six *S. aureus* and three *S. epidermidis* strains. Three *S. aureus* clinical strains producing a polysaccharide matrix and one protein-dependent biofilm strain from bovine origin were tested. Additionally, two *S. aureus* strains isolated from a food environment and three *S. epidermidis* of clinical origin, all of them carrying biofilm related genes, were included in the assay (Table 1). 24 h-old biofilms developed on 96-well polystyrene plates were treated with LysH5 (0.15 µM) and lysostaphin (0.2 µM) and biofilm removal was determined after 6 h of incubation. LysH5 showed a notable disrupting activity against biofilms of both *S. aureus* and *S. epidermidis* strains, as judged by the reduction of the attached bacterial counts by 1–3 log units per well (Fig. 1). The activity of this protein turned out to be higher than that of lysostaphin, with the exception of the biofilms developed by *S. aureus* 15981 and *S. aureus* ISP479r, against which lysostaphin was more effective. As expected, lysostaphin was quite ineffective against *S. epidermidis* biofilms, while LysH5 showed similar viable count reduction percentages against strains of both species.

The variability observed in the activity of LysH5 against the different biofilms might be determined by the susceptibility of the planktonic cells to LysH5 but also by the composition of the extracellular material which might impair or limit the access of LysH5 to the bacterial cells. However, we could not establish a correlation between the activity of LysH5 against each biofilm and the intrinsic susceptibility of each strain or the composition of the biofilm matrix. As shown in Table 2, the specific activity of LysH5 on exponentially growing *S. epidermidis* cells was rather low (1.5–3.8 ΔOD/mg min) in contrast to that shown by *S. aureus* cells (2.1–16.5 ΔOD/mg min). However, LysH5 removed staphylococcal biofilms regardless of the chemical composition of the extracellular matrix (polysaccharidic, protein or DNA based biofilms) (Table 3). Moreover, attached viable cells were recovered from the wells after a single exposure to LysH5 and the biofilm formation ability of these isolates was determined by crystal violet staining. Absorbance values identical to those of untreated strains were observed (Table S1). Thus, the surviving cells do not seem to have an increased biofilm-forming ability. Of note, a second LysH5 treatment resulted in the decrease of the staphylococcal populations to undetectable levels (<10 CFU/well) (Fig. 1). This suggests that the viable cells remaining attached to the well surface after the first LysH5 treatment were not persister cells as they remained susceptible to the endolysin.

**Table 2:** Sensitivity of staphylococcal strains to LysH5.

	Strain	Specific activity (ΔOD/mg min)	MIC (µM)
*S. aureus*	15981	13.4±0.8	0.1
	ISP479r	16.5±0.3	0.05
	V329	8.7±0.1	0.05
	132	14.3±0.4	0.1
	IPLA1	2.1±0.6	0.1
	IPLA16	3.0±0.1	0.05
	Newman	11.6±0.3	0.1
	SA113	12.1±0.2	0.1
*S. epidermidis*	B	3.8±0.1	0.05
	YLIC17	1.9±0.2	0.1
	DG2n	1.5±0.3	0.05

Specific activity of LysH5 against staphylococcal exponential cultures (ΔOD per mg of protein per min) and MIC values of LysH5 determined in staphylococcal broth cultures. The values are means ± standard deviations two biological experiments.

**Table 3:** Ability of specific treatments to remove biofilm formed by staphylococcal strains.

	Strain	Removed biofilm		
		NaIO_4_	Proteinase K	DNAse I
*S. aureus*	15981	+++	−	−
	ISP479r	++	++	+
	V329	−	+++	++
	132	+	+	+
	IPLA1	++	+	−
	IPLA16	++	++	−
*S. epidermidis*	B	+++	−	−
	YLIC17	+++	−	+
	DG2n	+++	−	+

Extracellular components of the biofilm matrix were estimated from the percentage of removed biofilm with specific treatments; sodium metaperiodate (NaIO_4_), proteinase K and DNAseI (+ = 30%; ++ = 30–70% and +++>70%).

### LTSEM supports biofilm disruption by LysH5

**Figure 2:**
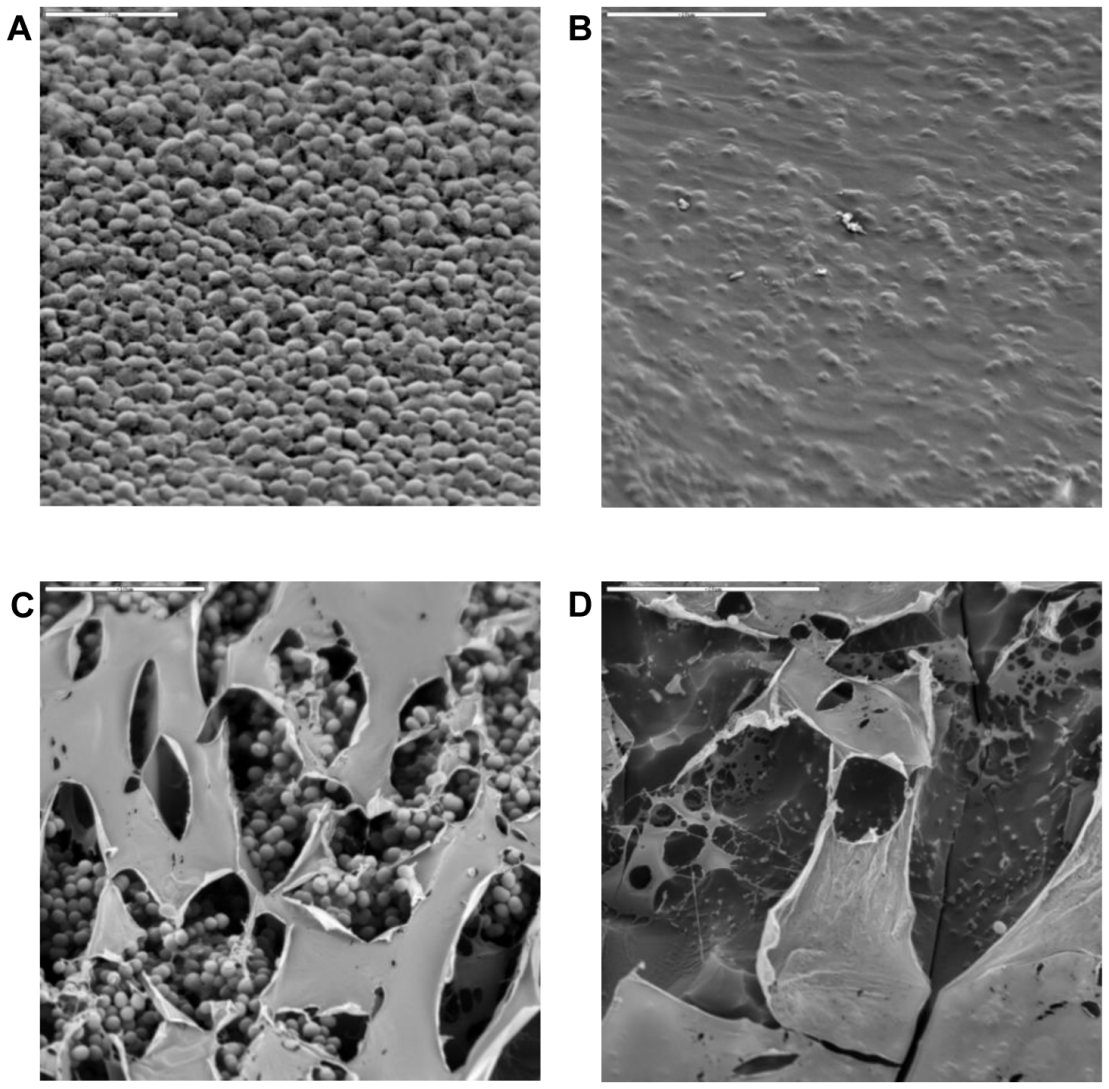
LTSEM micrographs of 24 h-old biofilms formed by *S. aureus* and *S. epidermidis*. Not treated biofilms of *S. aureus* 15981 and *S. epidermidis* YLIC17 are represented in A and C, respectively; biofilms after treatment with LysH5 are represented in B and D.

Electron micrographs reinforced our results as they showed unstructured biofilms as a consequence of the treatment with LysH5 (Fig. 2). In the case of *S. aureus* 15981, a polysaccharide-dependent biofilm producer, cells were arranged in layers and covered by a thin matrix that kept them attached to surface (Fig. 2A); after LysH5 treatment, no cells or extracellular material were observed (Fig. 2B). *S. epidermidis* YLIC17 biofilm structure showed cell aggregates producing a compact biofilm covered by a polysaccharidic layer (Fig. 2C) which was not removed by the endolysin treatment but whole cells were no longer present (Fig. 2D). This observation suggests that LysH5 was able to penetrate through the matrix structure.

### Sub-inhibitory concentrations of LysH5 do not induce staphylococcal biofilm and prevent its formation in some strains

**Figure 3:**
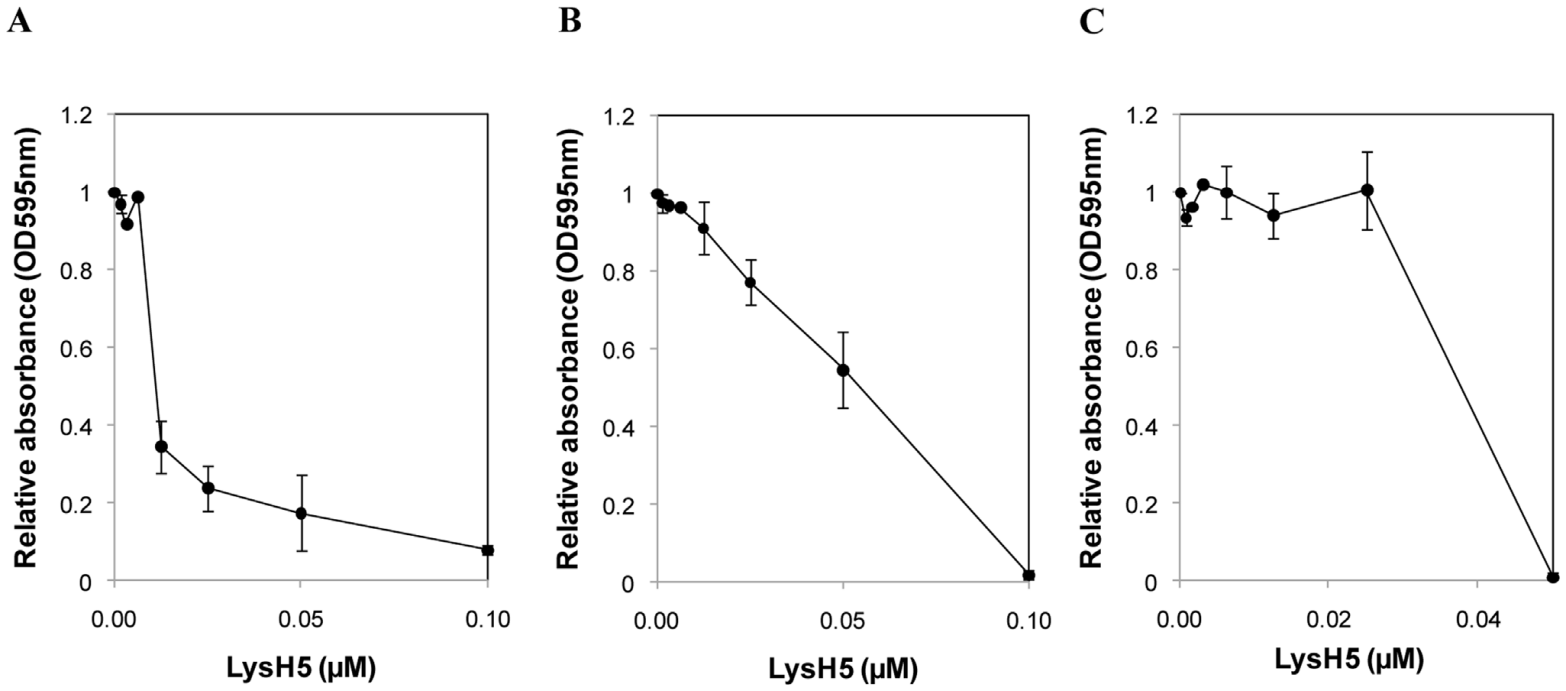
Behavior of 24 h-old biofilms formed by *S. aureus* and *S. epidermidis* strains, grown in the presence of sub-inhibitory concentrations of LysH5. Three strains were selected as representative of biofilm behavior (A) Prevention of the biofilm formation (*S. aureus* 15981); (B) Biofilm reduction (*S. aureus* ISP479r) and (C) no effect in biofilm formation (*S. aureus* IPLA16). Biofilm formation was expressed as relative absorbance (595 nm) of crystal violet stained cultures (treated/untreated cultures)(•). LysH5 concentration is expressed in µM. Each value is the mean ± standard deviation of two biological replicates.

To evaluate a putative inducing effect of LysH5 on biofilm formation, *S. aureus* strains were grown in the presence of LysH5 at concentrations lower than the MIC (Table 2) and checked for biofilm formation. As a control for antibiotic-induced biofilm, the strain *S. aureus* Newman was used [14]. As expected, a relative absorbance increase of 18-fold was observed for this strain treated with sub-inhibitory concentrations of methicillin and further stained with crystal violet. Significantly, induction in biofilm formation was also observed for strains *S. aureus* 132 and *S. aureus* IPLA1 (1.6 and 1.3 times, respectively) (Fig. S1). In the presence of sub-MIC concentrations of LysH5, bacterial growth was not inhibited and biofilm induction was not observed in any of the tested strains (Fig. S2). Interestingly, the staphylococcal strains behaved differently in the presence of sub-inhibitory LysH5 concentrations (Fig. 3). In fact, biofilm formation by *S. aureus* 15981, *S. aureus* 132 and *S. epidermidis* B was completely inhibited (Fig. 3A), while biofilm growth of *S. aureus* ISP479r, *S. aureus* V329, *S. aureus* IPLA1 and *S. epidermidis* YLIC17 was reduced, but not completely inhibited when growing at 0.25× to 0.5×MIC of LysH5 (Fig. 3B). For the remaining strains, *S. aureus* IPLA16, *S. aureus* Newman and *S. epidermidis* DG2n, no inhibition of biofilm formation was observed under sub-inhibitory concentrations (Fig. 3C).

### LysH5 is active against *S. aureus* planktonic persister cells

**Figure 4:**
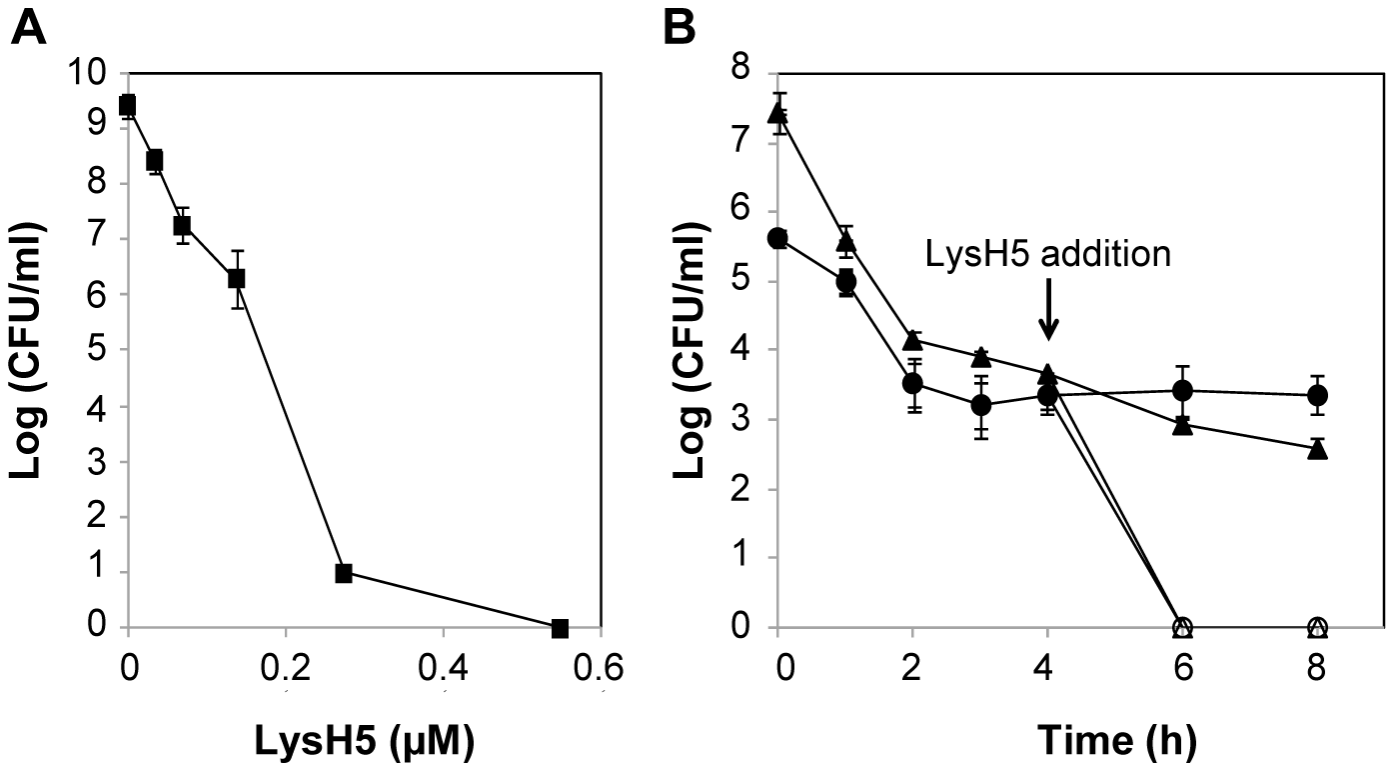
*S. aureus* persister cells selection and elimination by LysH5. (A) Activity of different concentrations of LysH5 against exponential cultures of *S. aureus* 15981. Means and standard deviations were calculated from two independent assays. Bacteria detection threshold (<10 CFU/ml). (B) Exponential cultures of *S. aureus* 113 treated with 2 µg/ml of rifampicin (•), and 3 µg/ml of ciprofloxacin (▴) for 4 h to select persister cells and subsequent treatment with 0.5 µM LysH5 (○ and Δ, respectively). Bacteria detection threshold (<10 CFU/ml). Each value represents the mean ± standard deviation of two biological replicates.

To assess if the treatment of staphylococcal planktonic cultures with LysH5 could select persister cells, *S. aureus* 15981 exponential cultures were treated with increasing LysH5 concentrations. Bacterial counts decreased as LysH5 concentration increased, and no surviving bacteria were detected at 0.5 µM endolysin (Fig. 4A). Similar results were obtained when *S. aureus* SA113 was treated with increasing concentrations of LysH5 (data not shown). To further confirm the effectiveness of LysH5, planktonic persister cells were isolated from *S. aureus* SA113 by treatment with either rifampicin or ciprofloxacin [35]. Fig. 4B shows the selection of persister cells by rifampicin and ciprofloxacin with the typical biphasic killing curve, and their further behavior after the addition of 0.5 µM LysH5. Indeed, no viable bacteria were detected after 2 h of incubation at 37°C, supporting the LysH5 lytic potential against this subpopulation of bacteria that can survive the antibiotic challenge.

## Discussion

The endolysin LysH5 has a remarkable activity to lyse *S. aureus* and *S. epidermidis* planktonic cells [31], which has been extended against staphylococcal biofilms in the current work. Low concentrations of LysH5 (0.15 µM) were effective to decrease viable bacteria inside biofilms formed by either *S. aureus* or *S. epidermidis* strains. No correlation was observed between the ability of LysH5 to remove biofilms and the origin of the strains (clinical, food), the extracellular matrix (polysaccharide, protein/DNA) or the peptidoglycan composition, although the latter could have an impact on LysH5 activity, as shown by the higher specific activity of LysH5 against *S. aureus* versus *S. epidermidis* planktonic cells [31] (Table 2). However, low diffusion of LysH5 inside biofilms might represent the main limitation to protein activity, as judged by the similar activity of LysH5 against *S. aureus* and *S. epidermidis* biofilms (Fig. 1).

Beyond the ability of endolysins to remove biofilms, the aim of this work was to study the effects of LysH5 on both biofilm formation and putative surviving cells after the LysH5 treatment. In this regard, we have determined the impact of the expected low diffusion of LysH5 through the extracellular material and the consequent sub-inhibitory concentration inside the biofilms. Our results showed no increase in biofilm formation at low concentrations of LysH5, neither in *S. aureus* nor in *S. epidermidis* strains. On the contrary, sub-inhibitory concentrations of many antibiotics have been reported to enhance the production of PIA in *S. epidermidis* biofilms [37] or extracellular DNA in *S. aureus* biofilms [14]. It is not totally clear which mechanism mediates biofilm induction by some antibiotics but it is thought to be a consequence of a global stress response [37]. Furthermore, we found that endolysin LysH5 was able to inhibit biofilm formation in some strains, even at 0.125× MIC. A similar effect was previously described in gallidermin, which represses transcription of genes involved in primary adhesion and exopolysaccharide production [19].

One of the main issues in biofilm control is the presence of persister cells, a small subpopulation of cells that spontaneously enter a dormant state and, consequently, survive bactericidal antibiotic treatment. Persisters have been found for almost every type of antibiotic tested so far [16]. They can reestablish the population once the stress is removed [38], and are behind recalcitrant chronic infections [39]. With this in mind, it is remarkable that no persister bacteria were detected in staphylococcal biofilms after LysH5-treatment. Surviving bacteria were still sensitive to the endolysin, confirming previous results of the lack of resistance after the exposure of *S. aureus* to LysH5 [33]. These results are also in accordance with the absence of reports on bacteria resistant to phage endolysins, despite several attempts to select them [40].

Keeping in mind that approximately 0.001–0.1% of cells of an isogenic bacterial population display tolerance to antibiotics [14], we also approached the selection of persister cells from exponential cultures. In a typical assay for selection of persister cells by antibiotics, the number of cells in the population is initially decreased by the antibiotic action, but in a second phase, despite increasing the time or the antibiotic concentration, a subpopulation of persister cells remain (biphasic curve) [41]. In a similar assay carried out with LysH5, a lack of persister bacteria after treatment of *S. aureus* exponential cultures with LysH5 was confirmed. The survival rates of bacteria treated with increasing concentrations of LysH5 failed to show a biphasic curve and no surviving bacteria were detected at high LysH5 concentration. In this regard, the fact that LysH5 is able to eliminate persister bacteria previously selected by two antibiotics, rifampicin and ciprofloxacin, is particularly interesting. As previously reported [35], treatment of *S. aureus* SA113 exponential cultures with 10×MIC ciprofloxacin and 100×MIC rifampicin resulted in a drug-tolerant population of about 10^3^ CFU/ml, which were further eliminated by LysH5. Our findings suggest that the endolysin target is accessible in a dormant state in contrast to antibiotics that need an active target only available in growing cells. Therefore, delivering endolysin LysH5 as adjuvant to some antibiotics would be beneficial in the treatment of chronic bacterial infections. Regarding the disinfection process of industrial food facilities, a two-step treatment could guarantee the removal of any persistent contamination and open the way to the use of LysH5 as part of the routine process exploiting putative synergistic action with other disinfectants/antimicrobials. Despite these promising results, the total duration of the two-step treatment (18 h) should be shortened, increasing the concentration of endolysin in order to achieve the desire efficacy, avoiding the interruption of the production in food industries. Moreover, our data should be confirmed in dynamic biofilm models, we provide additional proof of concept supporting the role of phage endolysins, not only as antibacterial agents against staphylococcal biofilms, but also as anti-persister agents, and therefore being an effective weapon to combat bacterial infections.

## Supporting Information

Figure S1 **Biofilms formed by** ***S. aureus*** **and** ***S. epidermidis*** **strains grown in the presence of sub-inhibitory concentrations of meticillin.** (A) Strains with antibiotic-induction of the biofilm; (B) strains with no effect in biofilm formation. Biofilm formation was expressed as relative absorbance (595 nm) of crystal violet stained cultures (treated/untreated cultures) (•). Each value is the mean ± standard deviation of two biological replicates.
(TIF)

Figure S2 **Biofilms formed by** ***S. aureus*** **and** ***S. epidermidis*** **strains grown in the presence of sub-inhibitory concentrations of LysH5.** (A) Strains showing prevention of the biofilm formation; (B) strains showing a biofilm reduction and (C) strains with no effect in biofilm formation. Biofilm formation was expressed as relative absorbance (595 nm) of crystal violet stained cultures (treated/untreated cultures) (•). Each value is the mean ± standard deviation of two biological replicates.
(TIF)

Table S1 **Biofilm formation ability of the** ***S. aureus and S. epidermidis*** **strains.** Comparison of 24 h biofilm growth using o/n cultures of the staphylococcal strains and o/n cultures of surviving cells recovered after biofilms LysH5 treatment. Values expressed as absorbance units (595 nm) of crystal violet stained cultures are the means ± standard deviations of two independent experiments.
(DOC)
