# Supplementary figures and images for: Effective Removal of Staphylococcal Biofilms by the Endolysin LysH5
Source: PLoS One. 2014 Sep 9;9(9):e107307. doi: 10.1371/journal.pone.0107307 (PMC4159335; doi:10.1371/journal.pone.0107307)

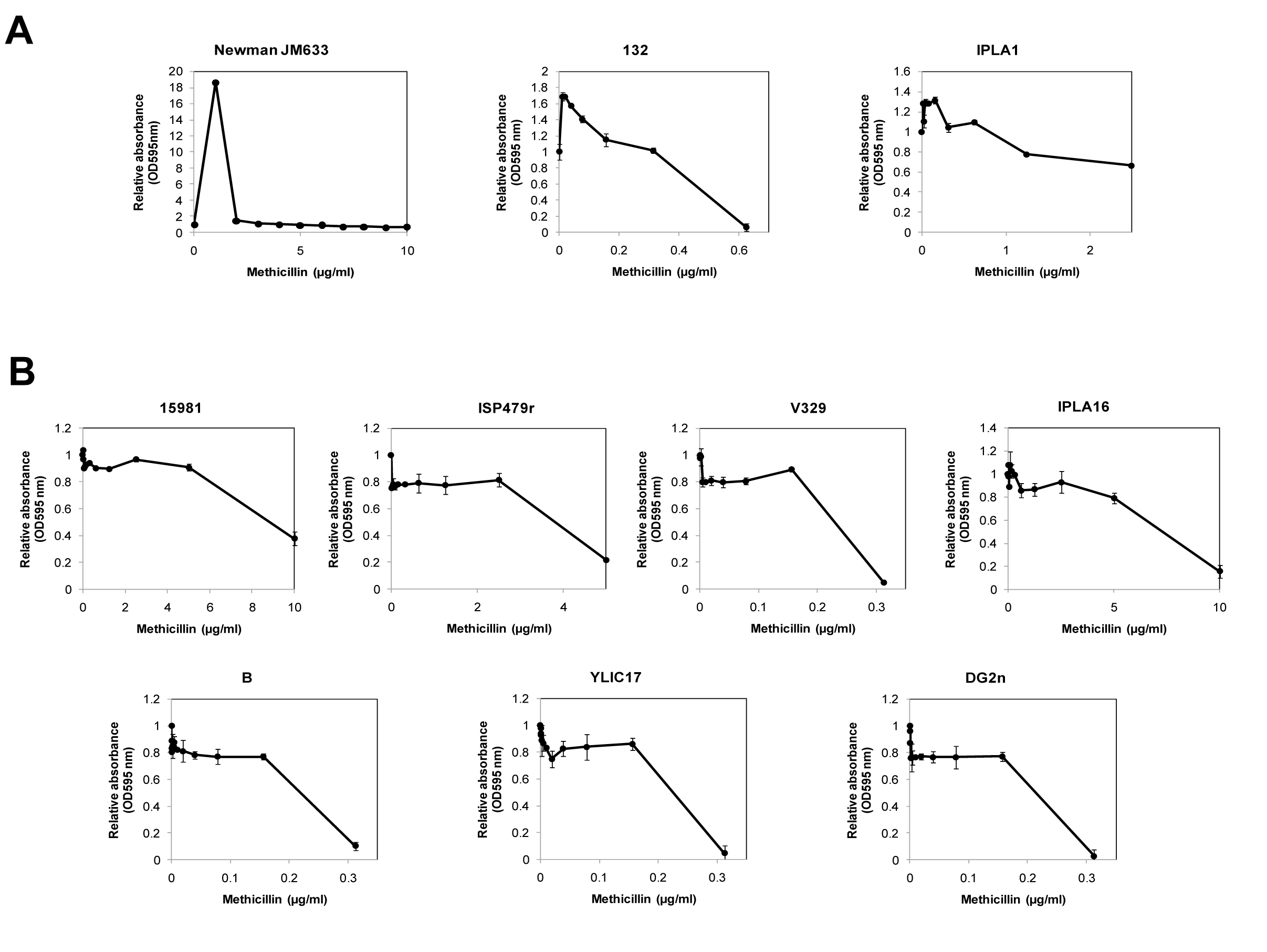

Supplement: Figure S1 — Biofilms formed by S. aureus and S. epidermidis strains grown in the presence of sub-inhibitory concentrations of meticillin. (A) Strains with antibiotic-induction of the biofilm; (B) strains with no effect in biofilm formation. Biofilm formation was expressed as relative absorbance (595 nm) of crystal violet stained cultures (treated/untreated cultures) (•). Each value is the mean ± standard deviation of two biological replicates. (TIF) [file pone.0107307.s001.tif]

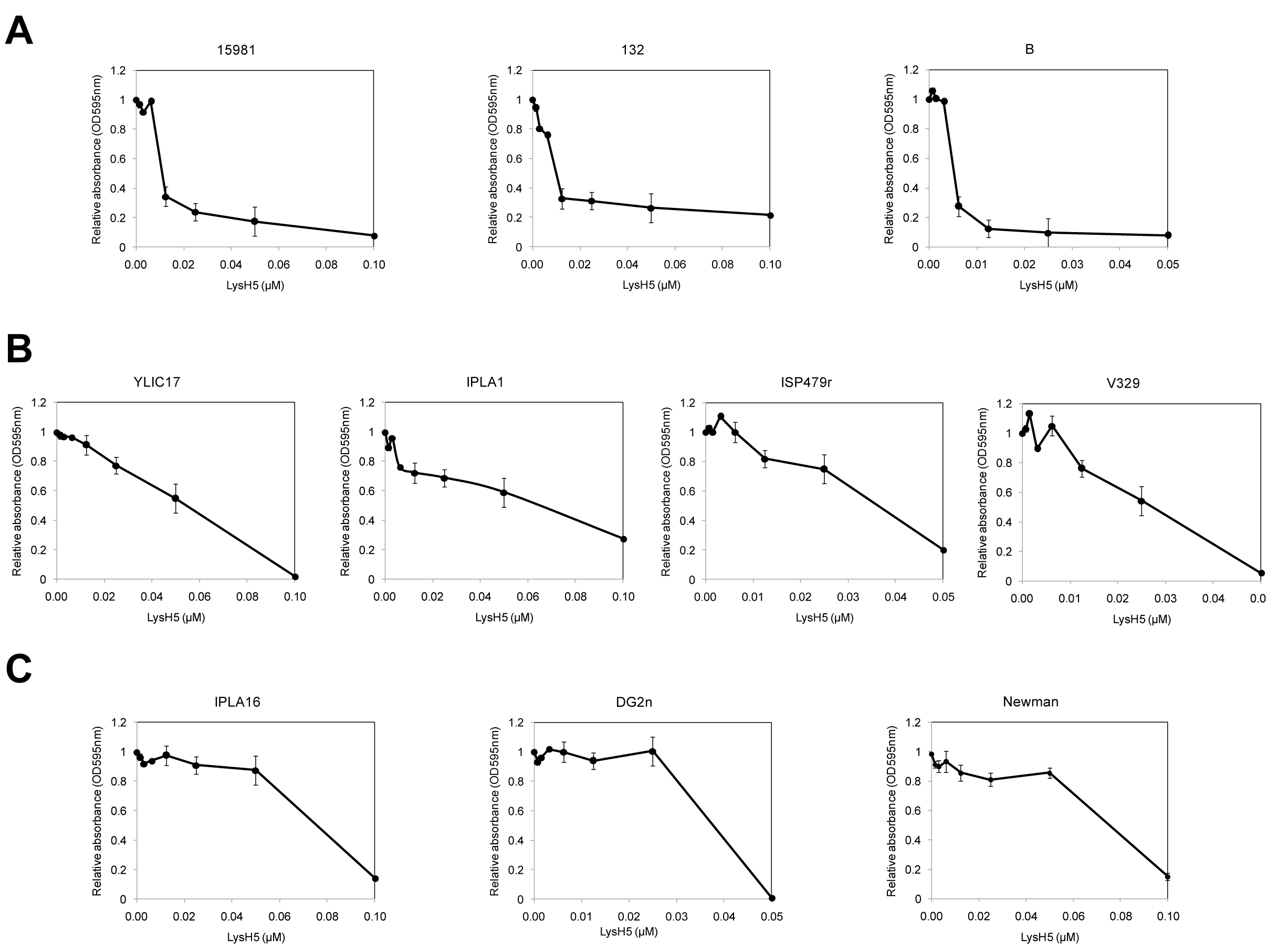

Supplement: Figure S2 — Biofilms formed by S. aureus and S. epidermidis strains grown in the presence of sub-inhibitory concentrations of LysH5. (A) Strains showing prevention of the biofilm formation; (B) strains showing a biofilm reduction and (C) strains with no effect in biofilm formation. Biofilm formation was expressed as relative absorbance (595 nm) of crystal violet stained cultures (treated/untreated cultures) (•). Each value is the mean ± standard deviation of two biological replicates. (TIF) [file pone.0107307.s002.tif]
