# Supplementary material for: Effective Removal of Staphylococcal Biofilms by the Endolysin LysH5
Source: PLoS One. 2014 Sep 9;9(9):e107307. doi: 10.1371/journal.pone.0107307 (PMC4159335; doi:10.1371/journal.pone.0107307)
Supplement: Table S1 — Biofilm formation ability of the S. aureus and S. epidermidis strains. Comparison of 24 h biofilm growth using o/n cultures of the staphylococcal strains and o/n cultures of surviving cells recovered after biofilms LysH5 treatment. Values expressed as absorbance units (595 nm) of crystal violet stained cultures are the means ± standard deviations of two independent experiments. (DOC) [file pone.0107307.s003.doc]

|  | **Strain** | **Biofilm formation ability*** | |
| --- | --- | --- | --- |
|  |  | **o/n cultures** | **Cells recovered from LysH5 treated biofilms** |
| *S. aureus* | 15981 | 9.3±0.3 | 10.2±0.7 |
|  | ISP479r | 1.7±0.2 | 1.8±0.1 |
|  | V329 | 11.4±0.3 | 12.2±0.4 |
|  | 132 | 1.7±0.3 | 2.0±0.2 |
|  | IPLA1 | 0.7±0.1 | 0.9±0.2 |
|  | IPLA16 | 1.1±0.1 | 1.0±0.1 |
| *S. epidermidis* | B | 14.3±0.2 | 14.0±0.2 |
|  | YLIC17 | 15.2±0.6 | 15.9±0.9 |
|  | DG2n | 16.9±0.5 | 16.9±0.4 |

**Table S1. Biofilm formation ability of the *S. aureus and S. epidermidis* strains.**Comparison of 24h-biofilms growth using o/n cultures of the staphylococcal strains and o/n cultures of surviving cells recovered after biofilms LysH5 treatment.

(*) Differences not statistically significant (ANOVA; *P*>0.05).
